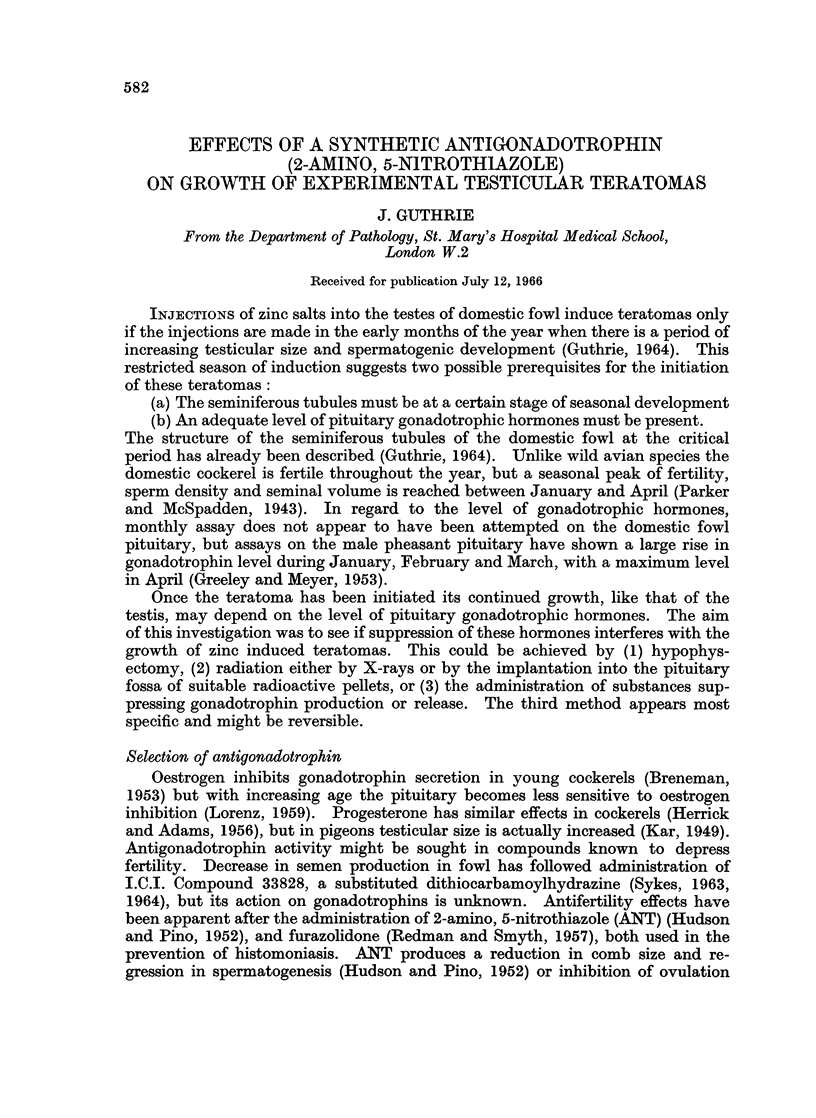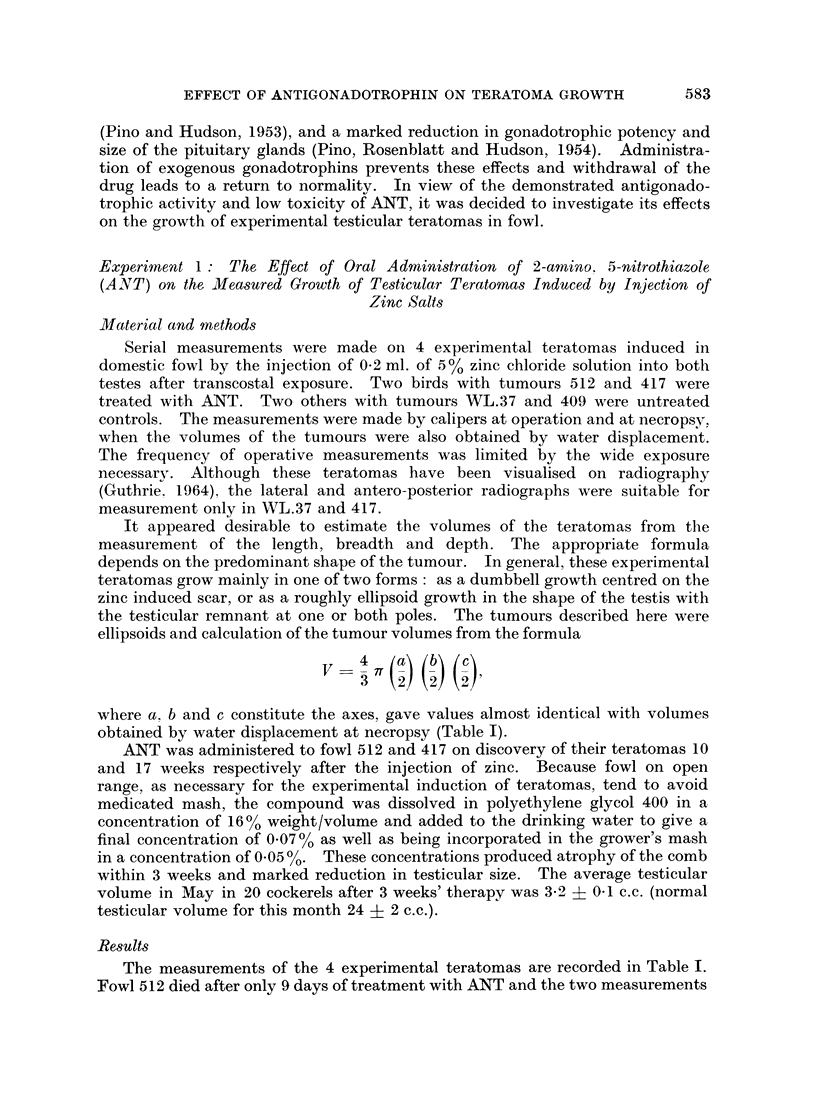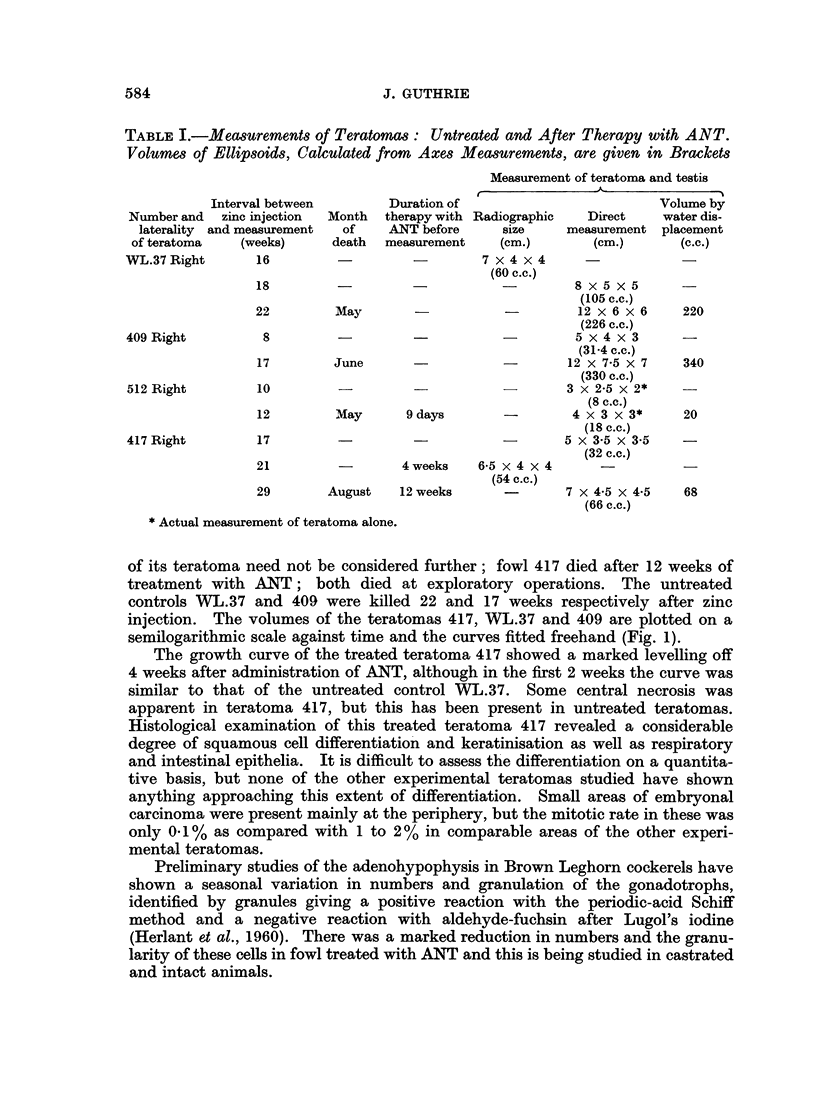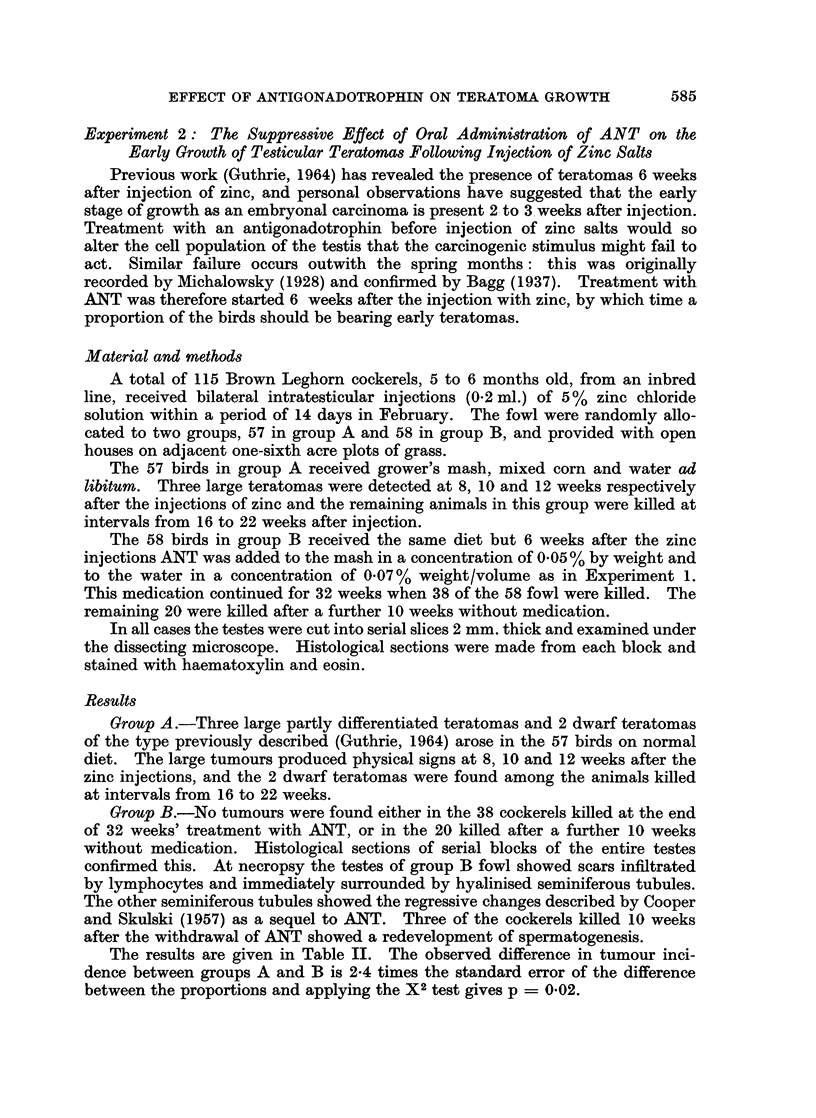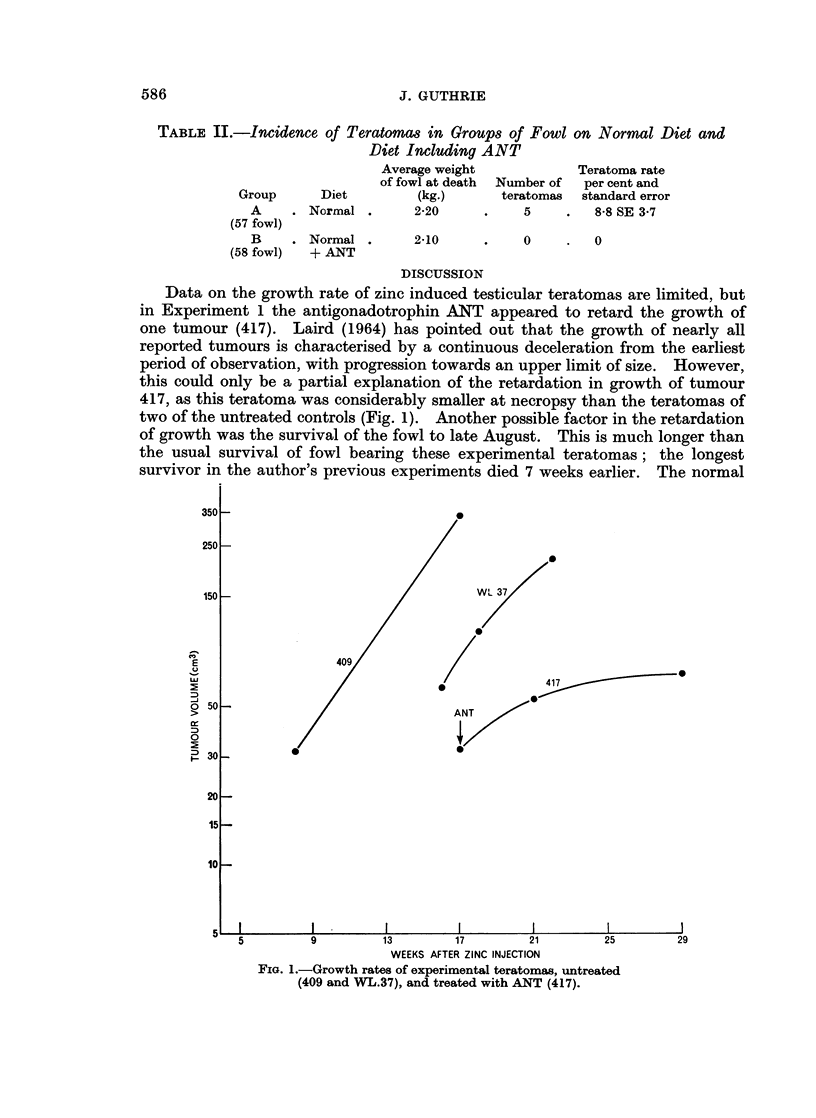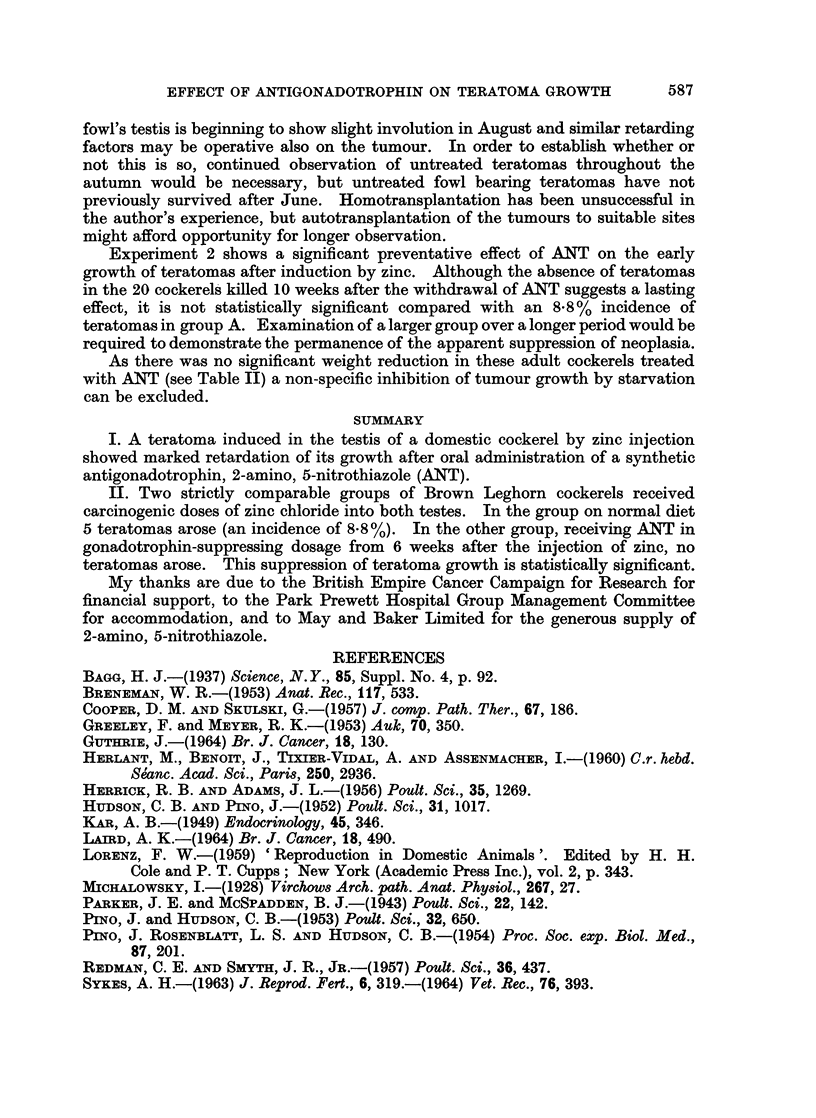# Effects of a synthetic antigonadotrophin (2-amino,5-nitrothiazole) on growth of experimental testicular teratomas.

**DOI:** 10.1038/bjc.1966.72

**Published:** 1966-09

**Authors:** J. Guthrie


### EFFECTS OF A SYNTHETIC ANTIGONADOTROPHIN (2-AMINO, 5-NITROTHIAZOLE) ON GROWTH OF EXPERIMENTAL TESTICULAR TERATOMAS

J. GUTHRIE

*From the Department of Pathology, St. Mary's Hospital Medical School, London W.2*

Received for publication July 12, 1966

INJECTIONS of zinc salts into the testes of domestic fowl induce teratomas only if the injections are made in the early months of the year when there is a period of increasing testicular size and spermatogenic development (Guthrie, 1964). This restricted season of induction suggests two possible prerequisites for the initiation of these teratomas :

(a) The seminiferous tubules must be at a certain stage of seasonal development

(b) An adequate level of pituitary gonadotrophic hormones must be present.

The structure of the seminiferous tubules of the domestic fowl at the critical period has already been described (Guthrie, 1964). Unlike wild avian species the domestic cockerel is fertile throughout the year, but a seasonal peak of fertility, sperm density and seminal volume is reached between January and April (Parker and McSpadden, 1943). In regard to the level of gonadotrophic hormones, monthly assay does not appear to have been attempted on the domestic fowl pituitary, but assays on the male pheasant pituitary have shown a large rise in gonadotrophin level during January, February and March, with a maximum level in April (Greeley and Meyer, 1953).

Once the teratoma has been initiated its continued growth, like that of the testis, may depend on the level of pituitary gonadotrophic hormones. The aim of this investigation was to see if suppression of these hormones interferes with the growth of zinc induced teratomas. This could be achieved by (1) hypophysectomy, (2) radiation either by X-rays or by the implantation into the pituitary fossa of suitable radioactive pellets, or (3) the administration of substances suppressing gonadotrophin production or release. The third method appears most specific and might be reversible.

### *Selection of antigonadotrophin*

Oestrogen inhibits gonadotrophin secretion in young cockerels (Breneman, 1953) but with increasing age the pituitary becomes less sensitive to oestrogen inhibition (Lorenz, 1959). Progesterone has similar effects in cockerels (Herrick and Adams, 1956), but in pigeons testicular size is actually increased (Kar, 1949). Antigonadotrophin activity might be sought in compounds known to depress fertility. Decrease in semen production in fowl has followed administration of I.C.I. Compound 33828, a substituted dithiocarbamoylhydrazine (Sykes, 1963, 1964), but its action on gonadotrophins is unknown. Antifertility effects have been apparent after the administration of 2-amino, 5-nitrothiazole (ANT) (Hudson and Pino, 1952), and furazolidone (Redman and Smyth, 1957), both used in the prevention of histomoniasis. ANT produces a reduction in comb size and regression in spermatogenesis (Hudson and Pino, 1952) or inhibition of ovulation

(Pino and Hudson, 1953), and a marked reduction in gonadotrophic potency and size of the pituitary glands (Pino, Rosenblatt and Hudson, 1954). Administration of exogenous gonadotrophins prevents these effects and withdrawal of the drug leads to a return to normality. In view of the demonstrated antigonadotrophic activity and low toxicity of ANT, it was decided to investigate its effects on the growth of experimental testicular teratomas in fowl.

*Experiment 1: The Effect of Oral Administration of 2-amino, 5-nitrothiazole (ANT) on the Measured Growth of Testicular Teratomas Induced by Injection of Zinc Salts*

*Material and methods*

Serial measurements were made on 4 experimental teratomas induced in domestic fowl by the injection of 0·2 ml. of 5% zinc chloride solution into both testes after transcostal exposure. Two birds with tumours 512 and 417 were treated with ANT. Two others with tumours WL.37 and 409 were untreated controls. The measurements were made by calipers at operation and at necropsy, when the volumes of the tumours were also obtained by water displacement. The frequency of operative measurements was limited by the wide exposure necessary. Although these teratomas have been visualised on radiography (Guthrie, 1964), the lateral and antero-posterior radiographs were suitable for measurement only in WL.37 and 417.

It appeared desirable to estimate the volumes of the teratomas from the measurement of the length, breadth and depth. The appropriate formula depends on the predominant shape of the tumour. In general, these experimental teratomas grow mainly in one of two forms : as a dumbbell growth centred on the zinc induced scar, or as a roughly ellipsoid growth in the shape of the testis with the testicular remnant at one or both poles. The tumours described here were ellipsoids and calculation of the tumour volumes from the formula

$$V = \frac{4}{3}\pi \left(\frac{a}{2}\right)\left(\frac{b}{2}\right)\left(\frac{c}{2}\right),$$

where $a$, $b$ and $c$ constitute the axes, gave values almost identical with volumes obtained by water displacement at necropsy (Table I).

ANT was administered to fowl 512 and 417 on discovery of their teratomas 10 and 17 weeks respectively after the injection of zinc. Because fowl on open range, as necessary for the experimental induction of teratomas, tend to avoid medicated mash, the compound was dissolved in polyethylene glycol 400 in a concentration of 16% weight/volume and added to the drinking water to give a final concentration of 0·07% as well as being incorporated in the grower's mash in a concentration of 0·05%. These concentrations produced atrophy of the comb within 3 weeks and marked reduction in testicular size. The average testicular volume in May in 20 cockerels after 3 weeks' therapy was 3·2 ± 0·1 c.c. (normal testicular volume for this month 24 ± 2 c.c.).

*Results*

The measurements of the 4 experimental teratomas are recorded in Table I. Fowl 512 died after only 9 days of treatment with ANT and the two measurements

TABLE I.—*Measurements of Teratomas : Untreated and After Therapy with ANT. Volumes of Ellipsoids, Calculated from Axes Measurements, are given in Brackets*

| Number and laterality of teratoma | Interval between zinc injection and measurement (weeks) | Month of death | Duration of therapy with ANT before measurement | Measurement of teratoma and testis: Radiographic size (cm.) | Direct measurement (cm.) | Volume by water displacement (c.c.) |
|---|---|---|---|---|---|---|
| WL.37 Right | 16 | — | — | 7 × 4 × 4 (60 c.c.) | — | — |
| | 18 | — | — | — | 8 × 5 × 5 (105 c.c.) | — |
| | 22 | May | — | — | 12 × 6 × 6 (226 c.c.) | 220 |
| 409 Right | 8 | — | — | — | 5 × 4 × 3 (31·4 c.c.) | — |
| | 17 | June | — | — | 12 × 7·5 × 7 (330 c.c.) | 340 |
| 512 Right | 10 | — | — | — | 3 × 2·5 × 2* (8 c.c.) | — |
| | 12 | May | 9 days | — | 4 × 3 × 3* (18 c.c.) | 20 |
| 417 Right | 17 | — | — | — | 5 × 3·5 × 3·5 (32 c.c.) | — |
| | 21 | — | 4 weeks | 6·5 × 4 × 4 (54 c.c.) | — | — |
| | 29 | August | 12 weeks | — | 7 × 4·5 × 4·5 (66 c.c.) | 68 |

* Actual measurement of teratoma alone.

of its teratoma need not be considered further ; fowl 417 died after 12 weeks of treatment with ANT ; both died at exploratory operations. The untreated controls WL.37 and 409 were killed 22 and 17 weeks respectively after zinc injection. The volumes of the teratomas 417, WL.37 and 409 are plotted on a semilogarithmic scale against time and the curves fitted freehand (Fig. 1).

The growth curve of the treated teratoma 417 showed a marked levelling off 4 weeks after administration of ANT, although in the first 2 weeks the curve was similar to that of the untreated control WL.37. Some central necrosis was apparent in teratoma 417, but this has been present in untreated teratomas. Histological examination of this treated teratoma 417 revealed a considerable degree of squamous cell differentiation and keratinisation as well as respiratory and intestinal epithelia. It is difficult to assess the differentiation on a quantitative basis, but none of the other experimental teratomas studied have shown anything approaching this extent of differentiation. Small areas of embryonal carcinoma were present mainly at the periphery, but the mitotic rate in these was only 0·1% as compared with 1 to 2% in comparable areas of the other experimental teratomas.

Preliminary studies of the adenohypophysis in Brown Leghorn cockerels have shown a seasonal variation in numbers and granulation of the gonadotrophs, identified by granules giving a positive reaction with the periodic-acid Schiff method and a negative reaction with aldehyde-fuchsin after Lugol's iodine (Herlant *et al.*, 1960). There was a marked reduction in numbers and the granularity of these cells in fowl treated with ANT and this is being studied in castrated and intact animals.

*Experiment 2: The Suppressive Effect of Oral Administration of ANT on the Early Growth of Testicular Teratomas Following Injection of Zinc Salts*

Previous work (Guthrie, 1964) has revealed the presence of teratomas 6 weeks after injection of zinc, and personal observations have suggested that the early stage of growth as an embryonal carcinoma is present 2 to 3 weeks after injection. Treatment with an antigonadotrophin before injection of zinc salts would so alter the cell population of the testis that the carcinogenic stimulus might fail to act. Similar failure occurs outwith the spring months: this was originally recorded by Michalowsky (1928) and confirmed by Bagg (1937). Treatment with ANT was therefore started 6 weeks after the injection with zinc, by which time a proportion of the birds should be bearing early teratomas.

*Material and methods*

A total of 115 Brown Leghorn cockerels, 5 to 6 months old, from an inbred line, received bilateral intratesticular injections (0·2 ml.) of 5% zinc chloride solution within a period of 14 days in February. The fowl were randomly allocated to two groups, 57 in group A and 58 in group B, and provided with open houses on adjacent one-sixth acre plots of grass.

The 57 birds in group A received grower's mash, mixed corn and water *ad libitum*. Three large teratomas were detected at 8, 10 and 12 weeks respectively after the injections of zinc and the remaining animals in this group were killed at intervals from 16 to 22 weeks after injection.

The 58 birds in group B received the same diet but 6 weeks after the zinc injections ANT was added to the mash in a concentration of 0·05% by weight and to the water in a concentration of 0·07% weight/volume as in Experiment 1. This medication continued for 32 weeks when 38 of the 58 fowl were killed. The remaining 20 were killed after a further 10 weeks without medication.

In all cases the testes were cut into serial slices 2 mm. thick and examined under the dissecting microscope. Histological sections were made from each block and stained with haematoxylin and eosin.

*Results*

*Group A*.—Three large partly differentiated teratomas and 2 dwarf teratomas of the type previously described (Guthrie, 1964) arose in the 57 birds on normal diet. The large tumours produced physical signs at 8, 10 and 12 weeks after the zinc injections, and the 2 dwarf teratomas were found among the animals killed at intervals from 16 to 22 weeks.

*Group B*.—No tumours were found either in the 38 cockerels killed at the end of 32 weeks' treatment with ANT, or in the 20 killed after a further 10 weeks without medication. Histological sections of serial blocks of the entire testes confirmed this. At necropsy the testes of group B fowl showed scars infiltrated by lymphocytes and immediately surrounded by hyalinised seminiferous tubules. The other seminiferous tubules showed the regressive changes described by Cooper and Skulski (1957) as a sequel to ANT. Three of the cockerels killed 10 weeks after the withdrawal of ANT showed a redevelopment of spermatogenesis.

The results are given in Table II. The observed difference in tumour incidence between groups A and B is 2·4 times the standard error of the difference between the proportions and applying the $X^2$ test gives $p = 0{\cdot}02$.

TABLE II.—*Incidence of Teratomas in Groups of Fowl on Normal Diet and Diet Including ANT*

| Group | Diet | Average weight of fowl at death (kg.) | Number of teratomas | Teratoma rate per cent and standard error |
|---|---|---|---|---|
| A (57 fowl) | Normal | 2·20 | 5 | 8·8 SE 3·7 |
| B (58 fowl) | Normal + ANT | 2·10 | 0 | 0 |

DISCUSSION

Data on the growth rate of zinc induced testicular teratomas are limited, but in Experiment 1 the antigonadotrophin ANT appeared to retard the growth of one tumour (417). Laird (1964) has pointed out that the growth of nearly all reported tumours is characterised by a continuous deceleration from the earliest period of observation, with progression towards an upper limit of size. However, this could only be a partial explanation of the retardation in growth of tumour 417, as this teratoma was considerably smaller at necropsy than the teratomas of two of the untreated controls (Fig. 1). Another possible factor in the retardation of growth was the survival of the fowl to late August. This is much longer than the usual survival of fowl bearing these experimental teratomas; the longest survivor in the author's previous experiments died 7 weeks earlier. The normal

FIG. 1.—Growth rates of experimental teratomas, untreated (409 and WL.37), and treated with ANT (417).

fowl's testis is beginning to show slight involution in August and similar retarding factors may be operative also on the tumour. In order to establish whether or not this is so, continued observation of untreated teratomas throughout the autumn would be necessary, but untreated fowl bearing teratomas have not previously survived after June. Homotransplantation has been unsuccessful in the author's experience, but autotransplantation of the tumours to suitable sites might afford opportunity for longer observation.

Experiment 2 shows a significant preventative effect of ANT on the early growth of teratomas after induction by zinc. Although the absence of teratomas in the 20 cockerels killed 10 weeks after the withdrawal of ANT suggests a lasting effect, it is not statistically significant compared with an 8·8% incidence of teratomas in group A. Examination of a larger group over a longer period would be required to demonstrate the permanence of the apparent suppression of neoplasia.

As there was no significant weight reduction in these adult cockerels treated with ANT (see Table II) a non-specific inhibition of tumour growth by starvation can be excluded.

### SUMMARY

I. A teratoma induced in the testis of a domestic cockerel by zinc injection showed marked retardation of its growth after oral administration of a synthetic antigonadotrophin, 2-amino, 5-nitrothiazole (ANT).

II. Two strictly comparable groups of Brown Leghorn cockerels received carcinogenic doses of zinc chloride into both testes. In the group on normal diet 5 teratomas arose (an incidence of 8·8%). In the other group, receiving ANT in gonadotrophin-suppressing dosage from 6 weeks after the injection of zinc, no teratomas arose. This suppression of teratoma growth is statistically significant.

My thanks are due to the British Empire Cancer Campaign for Research for financial support, to the Park Prewett Hospital Group Management Committee for accommodation, and to May and Baker Limited for the generous supply of 2-amino, 5-nitrothiazole.

### REFERENCES

Bagg, H. J.—(1937) *Science, N.Y.*, **85**, Suppl. No. 4, p. 92.
Breneman, W. R.—(1953) *Anat. Rec.*, **117**, 533.
Cooper, D. M. and Skulski, G.—(1957) *J. comp. Path. Ther.*, **67**, 186.
Greeley, F. and Meyer, R. K.—(1953) *Auk*, **70**, 350.
Guthrie, J.—(1964) *Br. J. Cancer*, **18**, 130.
Herlant, M., Benoit, J., Tixier-Vidal, A. and Assenmacher, I.—(1960) *C.r. hebd. Séanc. Acad. Sci., Paris*, **250**, 2936.
Herrick, R. B. and Adams, J. L.—(1956) *Poult. Sci.*, **35**, 1269.
Hudson, C. B. and Pino, J.—(1952) *Poult. Sci.*, **31**, 1017.
Kar, A. B.—(1949) *Endocrinology*, **45**, 346.
Laird, A. K.—(1964) *Br. J. Cancer*, **18**, 490.
Lorenz, F. W.—(1959) 'Reproduction in Domestic Animals'. Edited by H. H. Cole and P. T. Cupps; New York (Academic Press Inc.), vol. 2, p. 343.
Michalowsky, I.—(1928) *Virchows Arch. path. Anat. Physiol.*, **267**, 27.
Parker, J. E. and McSpadden, B. J.—(1943) *Poult. Sci.*, **22**, 142.
Pino, J. and Hudson, C. B.—(1953) *Poult. Sci.*, **32**, 650.
Pino, J. Rosenblatt, L. S. and Hudson, C. B.—(1954) *Proc. Soc. exp. Biol. Med.*, **87**, 201.
Redman, C. E. and Smyth, J. R., Jr.—(1957) *Poult. Sci.*, **36**, 437.
Sykes, A. H.—(1963) *J. Reprod. Fert.*, **6**, 319.—(1964) *Vet. Rec.*, **76**, 393.